# Early endosome autoantigen 1 regulates IL-1β release upon caspase-1 activation independently of gasdermin D membrane permeabilization

**DOI:** 10.1038/s41598-019-42298-4

**Published:** 2019-04-08

**Authors:** Alberto Baroja-Mazo, Vincent Compan, Fátima Martín-Sánchez, Ana Tapia-Abellán, Isabelle Couillin, Pablo Pelegrín

**Affiliations:** 1 Inflammation and Experimental Surgery Unit, Biomedical Research Institute of Murcia IMIB-Arrixaca, Clinical University Hospital Virgen de la Arrixaca, 30120 Murcia, Spain; 2 Institute of Functional Genomics, Labex ICST; INSERM U661, CNRS UMR5203, University of Montpellier.141, 34094 Montpellier cedex 5, France; 3 Molecular and Experimental Immunology and Neurogenetics, NEM, CNRS, UMR7355, University of Orleans, Orleans, 45071 France

## Abstract

Unconventional protein secretion represents an important process of the inflammatory response. The release of the pro-inflammatory cytokine interleukin (IL)-1β which burst during pyroptosis as a consequence of gasdermin D plasma membrane pore formation, can also occur through other unconventional secretion pathways dependent on caspase-1 activation. However, how caspase-1 mediates cytokine release independently of gasdermin D remains poorly understood. Here we show that following caspase-1 activation by different inflammasomes, caspase-1 cleaves early endosome autoantigen 1 (EEA1) protein at Asp^127/132^. Caspase-1 activation also results in the release of the endosomal EEA1 protein in a gasdermin D-independent manner. EEA1 knock-down results in adecreased release of caspase-1 and IL-1β, but the pyroptotic release of other inflammasome components and lactate dehydrogenase was not affected. This study shows how caspase-1 control the release of EEA1 and IL-1β in a pyroptotic-independent manner.

## Introduction

Caspase-1 is a protease that controls the release of different leaderless proteins via unconventional protein secretion, including its own release^1^. Caspase-1 secretome includes the well-characterized pro-inflammatory cytokine interleukin (IL)-1β. In addition to IL-1β, other cytokines, growth factors and intracellular proteins, including IL-18, IL-1β, high-mobility group protein B, fibroblast growth factors, or different redox proteins, are released via unconventional secretion^2–6^. Caspase-1 is activated by intracellular protein oligomers named inflammasomes, which are mainly formed by innate immune receptors of the Nucleotide oligomerization domain and Leucine rich repeat receptors (NLR) family^7^. Activation of caspase-1 induces pyroptotic cell death by cleavage of gasdermin D and its insertion in the plasma membrane causing pores, ultimately leading to the release of cytokines and inflammasome oligomeric aggregates^8–12^. Intracellular protein release induced by caspase-1 could also occur independently of cell death^6,9^, such as the case for monocytes^13^, and for example release of IL-1β could occur through microvesicle shedding, lysosomal/autophagosome release, exosome budding or gasdermin D pores in the absence of cell death^14–19^.

The early endosomal autoantigen 1 (EEA1) is an essential protein of the endosome docking and fusion machinery^20,21^. EEA1 protein contains a long helical coiled-coil central domain required for dimerization and two zinc-finger domains at its extremes, a C-terminal FYVE domain responsible for binding phospatidyl inositol-3-phospahte and a N-terminal C2H2 domain. EEA1 associates to small GTP-binding proteins of the Rab family^22^. These features confer membrane binding and fusion-inducing activities to EEA1, because once the endosome membrane associates to EEA1 it primes endosomes for subsequent fusion^23^. Here we found that, upon activation, caspase-1 cleaves EEA1 at Asp^127/132^ and induces the release EEA1 protein from the cell. The release of EEA1 was independent of gasdermin D and also occurs on the alternative NLRP3 activation pathway that resulted in a pyroptosis-independent release of IL-1β. Deficiency of EEA1 in macrophages resulted in a decrease of IL-1β release upon inflammasome activation, without affecting pyroptosis. EEA1 release into a pro-inflammatory environment with IL-1β could promote the initiation of autoimmune diseases.

## Results

### EEA1 is processed and released by caspase-1

**Figure 1:**
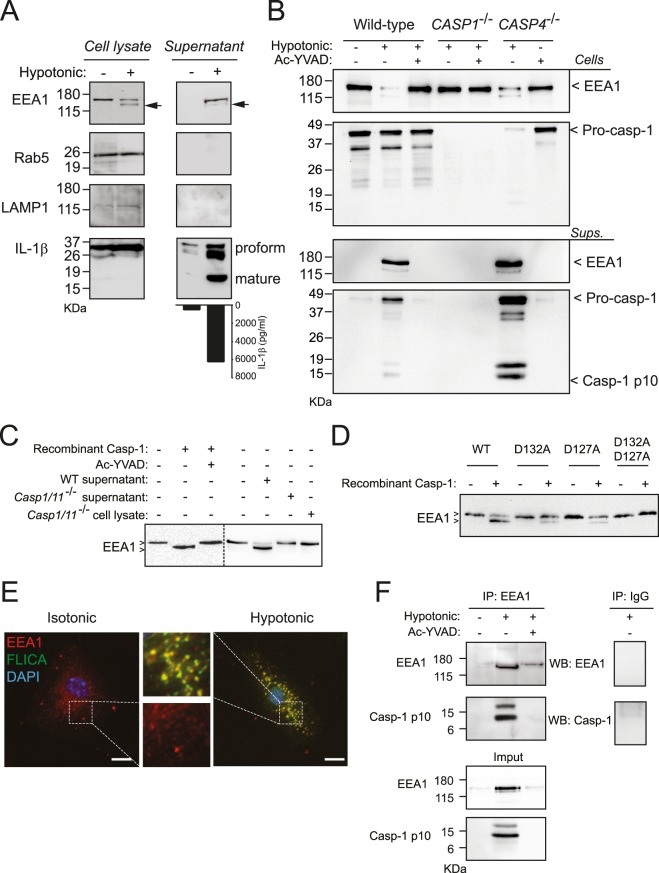
Caspase-1 induces the release of EEA1. (**A**) Canonical NLRP3 activation by hypotonic solution (90 mOsm, 1 h) in LPS-primed THP-1. Cell lysate and concentrated cell-free supernatant were analyzed by immunoblot for EEA1, Rab5, LAMP1 and IL-1β. Arrows denotes a smaller product of EEA1 upon hypotonic stimulation. IL-1β released to the extracellular medium was also detected by ELISA (bottom). (**B**) Immunoblot analysis of EEA1 and caspase-1 on cell lysate and cell-free supernatant from LPS-primed THP-1 (wild-type, *CASP1*^−/−^ or *CASP4*^−/−^) after hypotonic stimulation. Where indicated, Ac-YVAD was added to inhibit caspase-1. (**C**) *In vitro* processing assay for recombinant human EEA1 expressed in HEK293 cells and incubated with either recombinant caspase-1, or with concentrated supernatant or cell lysates from wild type (WT) or *Casp1/11*^−/−^ LPS-primed BMDM activated with nigericin. Where indicated, Ac-YVAD was added to inhibit caspase-1. Dashed line separates two different blots. (**D**) *In vitro* processing assay for recombinant human EEA1 WT, D132A, D127A, or D127A/D132A mutants expressed in HEK293 cells and incubated with recombinant caspase-1. (**E**) Representative high resolution deconvolved images of LPS-primed BMDMs after incubation with hypotonicity and stained with fluorescent probe for active caspase-1 (FLICA, green) and the endosome marker EEA1 (red) and nuclei (DAPI, blue); bar, 5 µm. (**F**) Co-immunoprecipitation of EEA1 and the p10 subunit of active caspase-1 in cell supernatants of LPS-primed THP-1 macrophages after NLRP3 stimulation with hypotonicity. Nitrocellulose membranes were cropped after protein blotting and then inmunodetection was carried out separately for each antibody. Complete membrane fragments are showed in all cases. Blots and pictures are representative of three to four independent experiments. ELISA data are presented as average ± sem from *n* = 3 independent experiments.

Macrophage swelling activates the NLR containing a Pyrin domain 3 (NLRP3) inflammasome and leads to the release of IL-1β upon caspase-1 activation^8,24,25^. Examination of THP-1 supernatants after NLRP3 inflammasome activation by hypotonic solution revealed the release of the endosome docking factor EEA1, but not Rab5 or the late-endosomal/lysosomal marker LAMP1 (Fig. 1A). Interestingly, hypotonic activation of the NLRP3 inflammasome resulted in the appearance of an additional lower molecular weight form of EEA1 in THP-1 cell lysates and supernatants, representing the 31.07 ± 15.24% and 7.97 ± 0.52% of the total, respectively (Fig. 1A). The processing and release of EEA1 was completely inhibited when the specific caspase-1 inhibitor Ac-YVAD was used or when THP-1 cells deficient on caspase-1, but not caspase-4, were used (Fig. 1B). Next, direct processing of EEA1 by caspase-1 was investigated by using an *in vitro* cleavage assay. We found that recombinant human caspase-1 was able to process recombinant EEA1 (Fig. 1C) and similarly, incubation of recombinant EEA1 with the supernatant from mouse bone marrow derived macrophages (BMDM) activated with nigericin, where native active caspase-1 is released^8^, also resulted in the cleavage of EEA1 (Fig. 1C). EEA1 processing was not observed using both supernatant or cell lysate from caspase-1/11-deficient mouse BMDMs (Fig. 1C). All these data show that EEA1 is a direct substrate of caspase-1. Bioinformatics analysis of human EEA1 sequence with ExPASy Peptide Cutter software^26^ and GraBCas software found three Asp at positions 132, 738 and 762 as the best candidates for caspase-1 cleavage, however ExPASy Peptide Cutter software and GraBCas software did not find any Asp in EEA1 as candidate for caspase-11 cleavage. Cleavage at Asp^132^ would result in a predicted EEA1 C-terminal fragment of 147 kDa (Supplementary Fig. S1), similar to the size found for EEA1 processing in cellular experiments and *in vitro* cleavage assays. Asp^132^ was confirmed as the cleavage site of caspase-1 after Edman N-terminus sequencing of EEA1 incubated with recombinant caspase-1. However, mutation of D132A result in a similar caspase-1 processing than wild-type EEA1 (Fig. 1D). EEA1 processing during apoptosis in different cell types has been reported to occur at Asp^127^and Asp^132 27^. Double EEA1 mutant D127A/D132A, but not single mutants, was not processed by caspase-1 (Fig. 1D).

Active caspase-1 localization was assessed in mouse BMDM using the fluorescent peptide FAM-YVAD-fmk FLICA^24,28^. FLICA green fluorescent signal was observed in macrophages after caspase-1 activation using hypotonicity, and was found to co-localize with EEA1 (R = 0.89, Fig. 1E, Supplementary Fig. S2A), whereas it poorly co-localized with the lysosomal marker LAMP1 (R = 0.62) or lysosomes stained with lysotracker (R = 0.56) (Supplementary Fig. S2B). In THP-1 supernatants, EEA1 appeared associated with the p10 subunit of active caspase-1 after NLRP3 inflammasome activation by hypotonicity (Fig. 1F). This interaction was absent when a caspase-1 inhibitor was used (Fig. 1F). This association suggest an interaction beyond caspase-1 activity, since proteases have a transient interaction with their substrates.

### Gasdermin D is dispensable for EEA1 release

**Figure 2:**
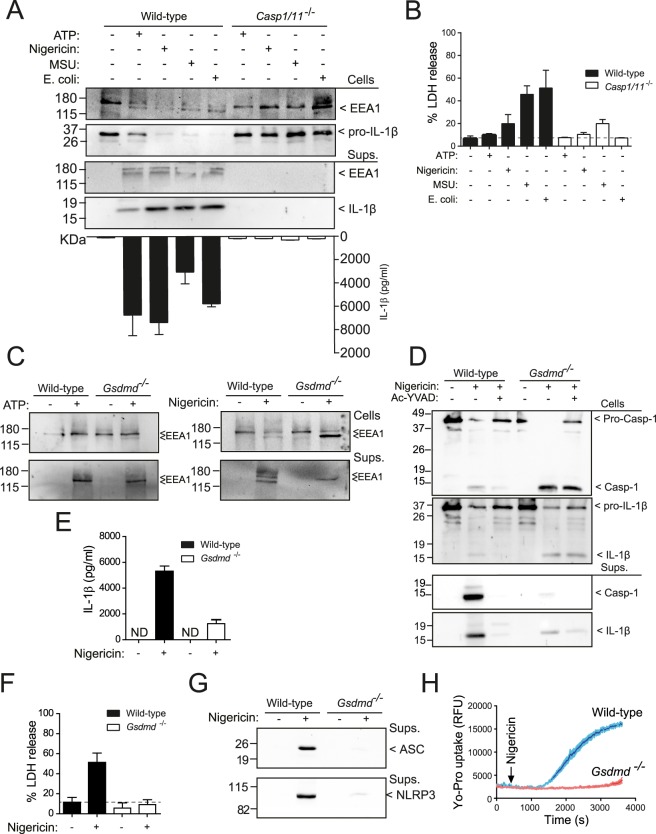
Gasdermin D pore formation and pyroptosis are not involved in EEA1 release. (**A**) Immunoblot analysis of EEA1 and IL-1β on cell lysate and cell-free supernatant from LPS-primed BMDMs from wild type or *Casp1/11*^−/−^ after stimulation with nigericin, ATP, *E*. *coli* or MSU crystals as indicated. IL-1β released to the extracellular medium was detected by ELISA (bottom). (**B**) LDH release from macrophages treated as in A. (**C**) Immunoblot analysis of EEA1 on cell lysate and cell-free supernatant from BMDMs wild-type and *Gsdmd*^−/−^ after nigericin stimulation. (**D**) Immunoblot analysis of caspase-1 and IL-1β on cell lysate and cell-free supernatant from wild-type or *Gsdmd*^−/−^ BMDMs after nigericin stimulation in the presence or absence of the caspase-1 inhibitor Ac-YVAD. (**E**,**F**) ELISA for released IL-1β (**E**) or LDH (**F**) from macrophages treated as in D. (**G**) Immunoblot analysis of ASC and NLRP3 on cell-free supernatant from macrophages stimulated as in D. (**H**) Yo-Pro uptake in macrophages treated as in D. Blots are representative of three to four independent experiments. Nitrocellulose membranes were cropped after protein blotting and then inmunodetection was carried out separately for each antibody. Complete blots for IL-1β and Caspase-1 are showed as supplementary. Data are presented as average ± sem from *n* = 3 independent experiments.

Activation of caspase-1 through the canonical NLRP3 inflammasome activation by ATP, nigericin, and uric acid crystals resulted in the processing and release of EEA1 (Fig. 2A), in parallel to caspase-1-dependent pyroptosis induction (Fig. 2B). Likewise, the non-canonical NLRP3 activation by live *E*. *coli* induced the release of EEA1 (Fig. 2A). In this pathway, intracellular LPS binds directly to caspase-11, which processes gasdermin D and evoke pyroptosis and subsequent activation of casapse-1 by NLRP3 inflammasome activation^29^. Moreover, activation of caspase-1 by other inflammasomes (NLRC4 or NLRP1) resulted in the processing and release of EEA1 (Supplementary Fig. 3A,B). Following ATP stimulation, the processing and release of EEA1 were completely inhibited in macrophages deficient for either P2X7 receptor, NLRP3 or ASC (Supplementary Fig. S3C). However, the release of EEA1 was occurring in gasdermin D-deficient macrophages (Fig. 2C). In the absence of gasdermin D we found a small release of IL-1β that was blocked after caspase-1 inhibition (Fig. 2D,E). However, deficiency of gasdermin D blocked pyroptosis, measured as the release of LDH or inflammasome proteins (NLRP3 and ASC) (Fig. 2F,G), and plasma membrane pore formation, measured by Yo-Pro uptake (Fig. 2H). This suggest that gasdermin D induced plasma membrane permeabilization and pyroptosis execution is not responsible for EEA1 release upon caspase-1 activation, but were responsible for the majority of IL-1β released from macrophages.

### EEA1 is released during the alternative NLRP3 activation pathway

**Figure 3:**
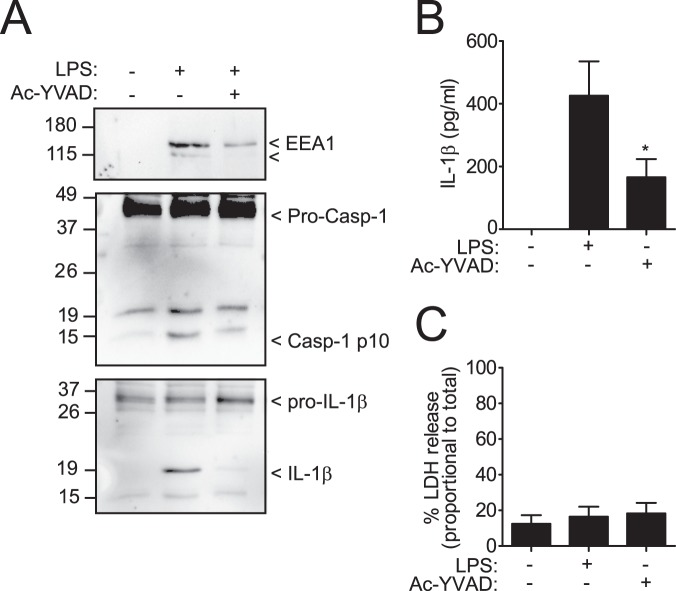
EEA1 is released during the alternative NLRP3 activation pathway in the absence of pyroptosis. (**A**) Immunoblot analysis of EEA1, caspase-1 and IL-1β on cell-free supernatant from human monocytes incubated during 4 h with 1 µg/ml of LPS. Blots are representative of three independent experiments. (**B**,**C**) ELISA for released IL-1β (**B**) or LDH (**C**) from isolated monocytes treated as in A. Nitrocellulose membranes were cropped after protein blotting and then inmunodetection was carried out separately for each antibody. Complete blots for IL-1β and Caspase-1 are showed as supplementary. Data are presented as average ± sem from *n* = 3 independent experiments. *p ≤ 0.05.

Human monocytes release IL-1β in the absence of pyroptotic cell death via the alternative activation pathway of NLRP3^13^. We found that upon alternative activation of NLRP3 in human monocytes there was processing and release of EEA1, and this process was blocked after caspase-1 inhibition (Fig. 3A). In parallel, mature IL-1β and caspase-1 were also released from monocytes (Fig. 3A,B). The release of cytokines and EEA1 from human monocytes was independent of pyroptosis, as the amount of extracellular LDH was not increased in supernatants (Fig. 3C). These data support the conclusion that EEA1 is released as consequence of caspase-1 activation in the absence of pyroptosis.

### EEA1 knock-down impaired IL-1β release but not pyroptotic cell death

**Figure 4:**
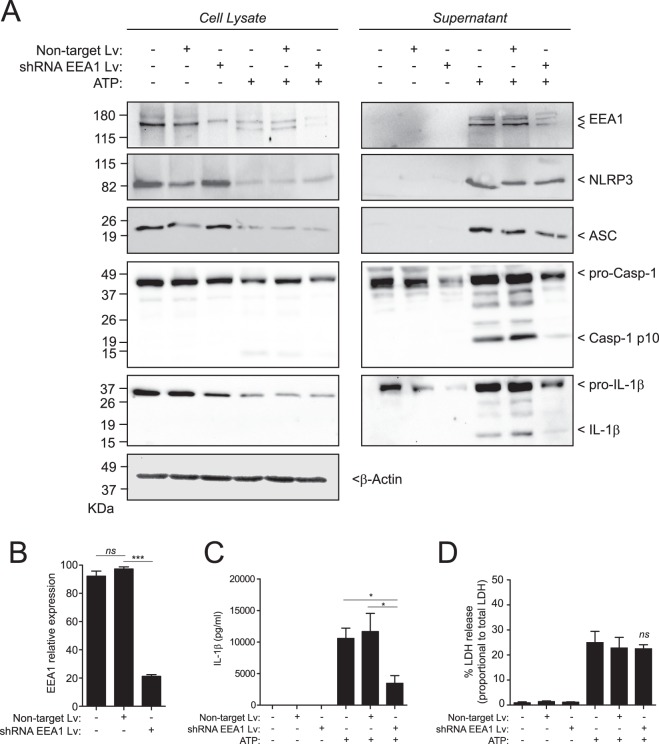
EEA1 knock down results in a decrease release of IL-1β from macrophages without affecting pyroptosis. (**A**) Immunoblot analysis of EEA1, NLRP3, ASC, caspase-1 and IL-1β on cell lysate and cell-free supernatant from BMDMs transduced with non-target control (Non-target Lv) or EEA1 specific shRNA lentiviral particles (shRNA EEA1 Lv), primed with LPS and activated with ATP as indicated. This figure is representative of three independent experiments. (**B**) Densitometry quantification of EEA1 immunoblots shown in A. Relative EEA1 expression is normalized to β-actin expression. (**C**,**D**) ELISA for released IL-1β (**C**) or LDH (**D**) from BMDMs treated as in A. Nitrocellulose membranes were cropped after protein blotting and then inmunostained separately for each protein. Complete blots for IL-1β ans Caspase-1 are showed as supplementary. Data are presented as average ± sem from *n* = 3 independent experiments. *ns*, not significant (*p* > 0.05); ***p < 0.0001; *p ≤ 0.05.

We next examined the effect of knocking-down EEA1 expression on IL-1β release. In cells transduced with lentivirus bearing a specific short hairpin RNA against *Eea1*, EEA1 expression was suppressed by 78.79 ± 2.11% compared to cells transduced with a lentivirus containing a non-target encoding sequence (Fig. 4A,B). Following canonical stimulation of NLRP3 inflammasome by ATP, EEA1 knock-down resulted in a decrease, but not completely blocking, of the release of IL-1β (45.46 ± 6.65% of release respect to non-target lentivirus) (Fig. 4A,C). However, the pyroptotic release of the inflammasome components NLRP3 and ASC (Fig. 4A) and LDH (Fig. 4D) was not affected by EEA1 silencing. Altogether the data show that caspase-1 could control the release of IL-1β by EEA1, independently and in parallel to gasdermin D induced pyroptotic cell death.

## Discussion

Gasdermin D pore formation on the plasma membrane and subsequent pyroptotic cell death upon caspase-1 activation has been described as a pathway for unconventional IL-1β release^11,12,30,31^. Oligomeric NLRP3 inflammasome particles containing ASC aggregates are also released from macrophages via pyroptotic cell death^8,25,32^. However, in some circumstances, gasdermin D pore formation has been found as responsible for IL-1β release in the absence of pyroptotic cell death^19^, explaining the release of IL-1β in the absence of cell death. Here we found that upon inflammasome activation, caspase-1 cleaves the endosomal docking-protein EEA1 at Asp^127/132^ and also induce its release. We cannot completely rule out the participation of caspase-11 in EEA1 processing after non-canonical inflammasome activation, however, human EEA1 sequence did not contain any Asp as candidate for caspase-11 cleavage, compared with the three Asp found as the best candidates for caspase-1 cleavage. The release of EEA1 was independent on gasdermin D and pyroptosis execution and account for the release of a pool of IL-1β. EEA1 release also occurs during the release of IL-1β in human monocytes in the absence of cell death upon the activation of the NLRP3 inflammasome alternative pathway.

Caspase-1 controls the unconventional release of the pro-inflammatory cytokine IL-1β as an important effector molecule of the inflammasome. Different unconventional secretion pathways have been described for IL-1β release, these involve intracellular vesicular trafficking (such as exocytosis of lysosomes or autophagosomes), the release of microvesicles and exosomes, or ultimately pyroptotic cell death induced by gasdermin D pore formation on the plasma membrane^9,10,12,31,33–36^. EEA1 represents an additional pathway to release IL-1β that is important for a pool of non-pyroptotic IL-1β release, as the release of IL-1β described in monocytes or neutrophils^13,37^. In fact, we found that the activation of the alternative pathway of NLRP3 in human monocytes also induces the release of EEA1. However, whether gasdermin D induced IL-1β release in the absence of pyroptosis^19^ applies to the alternative NLRP3 activation pathway found in human monocytes is not known. It seems unlikely that EEA1 could control the unconventional release mechanisms already described for IL-1β^33,34^. Multivesicular bodies are the origin of exosomes after fusing with plasma membrane^38,39^ and maturation of endosomes to multivesicular bodies, lysosomes or autophagolysosomes lack EEA1 protein association^40^. However, EEA1 could be controlling an endosomal-related release of IL-1β, since it is a key controller protein of endosomal docking, clustering and fusion via its association to small GTP-binding proteins of the Rab family^41–43^. Although we found EEA1 cleavage and release controlled by caspase-1, we cannot formally conclude that EEA1 cleavage by caspase-1 is dispensable for its release. The generation of macrophages carrying double EEA1 mutant D127A/D132A will be key to determine if caspase-1 processing of EEA1 is a key step for its release. However, due to the differential release of EEA1 forms (unprocessed *vs*. processed) from the different experiments presented in our study, we cannot conclude that EEA1 processing is a determinant step for its release.

During pyroptotic cell death, the release of IL-1β via gasdermin D pore formation and subsequent osmotic cell lysis^44^ overlaps to the release of IL-1β induced by EEA1. Silencing EEA1 expression decreases the release of mature IL-1β from macrophages, without affecting other markers of pyroptosis. Furthermore, deficiency of gasdermin D was not affecting EEA1 release upon caspase-1 activation. A recent gasdermin D-independent form of lytic cell death has been described upon NLRP3 inflammasome activation^45^, but EEA1 controlled IL-1β release will not explain this pathway as EEA1 did not affect cell lysis. Furthermore, Monteleone *et al*.^46^ have recently described that IL-1β maturation is sufficient for slow, gasdermin D-independent secretion of ectopic IL-1β from non-pyroptotic macrophages, but the speed of IL-1β release is boosted by inflammasome activation, via caspase-1 and gasdermin D. Mature IL-1β was found concentrated in plasma membrane ruffles enriched with phosphatidylinositol-4,5-biphosphate (PIP2), and this was a prerequisite for the slow and non-pyroptotic IL-1β secretion^46^. PIP2 is important for endocytic turnover trafficking and early endosomal homeostasis^47^, suggesting that it could be also important for the EEA1 controlled IL-1β release.

Autoantibodies to EEA1 have been described in patients with subacute cutaneous systemic lupus erythematosus, polyarthritis, rheumatoid arthritis, and neurological diseases^48,49^. It is poorly known how EEA1 could be exposed to the extracellular environment to induce autoantibody production. Although it is commonly assumed that these proteins could be present on apoptotic debris^50^, apoptosis and removal of apoptotic debris are immunologically silent process. Here we imply caspase-1 activation as controlling the release of EEA1 into a pro-inflammatory milieu that could favor autoantibodies generation against EEA1.

Taking together, our results point that caspase-1 induces the release of EEA1 independently and parallel to pyroptotic cell death, accounting for a pool of IL-1β release in the absence of gasdermin D plasma membrane permeabilization. During this process, EEA1 release could be important for the development of autoimmune diseases.

## Materials and Methods

### Animals

C57BL/6 (wild type, WT) mice were purchased from Harlan. P2X7 receptor-deficient mice (*P2rx7*^−/−^) were purchased from Jackson^51^, and *Gsdmd*^−/−^, double caspase-1/11-deficient (*Casp1/11*^−/−^), *Nlrp3*^−/−^ and *Pycard*^−/−^ mice^2,52,53^ were all on C57BL/6 background. For all experiments, mice between 8–10 weeks of age bred under SPF conditions were used in accordance with the University Hospital *Virgen Arrixaca* animal experimentation guidelines, and the Spanish national (RD 53/2013 and Law 6/2013) and EU (86/609/EEC and 2010/63/EU) legislation. According to legislation cited above, local ethics committee review approval is not needed, since mice were euthanized by CO_2_ inhalation and used to obtain bone marrow; no procedure was undertaken which compromised animal welfare (chapter 1, article 3 of RD 53/2013).

### Reagents

*E*. *coli* LPS O55:B5, DAPI, ATP, triton X-100, nigericin, anti-V5 antibody, PMA and puromycin resistant MISSION® shRNA Lentiviral Transduction Particles were from Sigma-Aldrich; Recombinant human caspase-1 and caspase-1 inhibitor Ac-YVAD-AOM were from Merk-Millipore; MSU crystals were from Enzo Life Sciences; flagellin from *S*. *typhimurium* from Invivogen; the receptor-binding protein protective antigen and metalloprotease lethal factor were from List Biological; the protein-delivery reagent PULSin was from PolyPlus Transfection;Yo-Pro-1 from Life Technologies. Antibodies for caspase-1 p10, IL-1β, ASC and LAMP1 were from Santa Cruz Biotechnology. Mouse monoclonal anti-NLRP3 was from AdipoGen. Mouse monoclonal anti-Rab5 and anti-EEA1 were from BD Biosciences. All HRP-conjugated secondary antibodies were from GE Healthcare and fluorescent-conjugated secondary antibodies were from Life Technologies.

### Cells, treatments and transfections

BMDMs were obtained from wild type or knockout mice as described^54^. HEK293 cells (CRL-11268; American Type Culture Collection) were maintained in DMEM:F12 (1:1) supplemented with 10% FCS and 2 mM Glutamax (Life Technologies). THP-1 wild-type (TIB202; American Type Culture Collection) and *Casp1*^−/−^ *or Casp4*^−/−^cells were kindly provided by Dr. V. Hornung^55^ and were maintained in RPMI supplemented with 10% FCS and 2 mM Glutamax. All cell lines were routinely tested for mycoplasma contamination with a Mycoplasma Detection Kit (Roche). THP-1 were treated with PMA (0.5 μM, 30 min). PMA-treated THP-1 and BMDMs were primed with LPS (1 μg/ml, 4 h) and subsequent canonical NLRP3 inflammasome activation was achieved with ATP (5 mM, 30 min), nigericin (10 μM, 30 min), MSU crystals (200 μg/ml, 16 h), hypotonic solution (90 mOsm, 1 h)^24^, live *E*. *coli* (20 MOI for 1 h, and then addition of 100 U/ml penicillin/streptomycin and incubate for further 16 h), anthrax lethal toxin (2.5 μg/ml receptor-binding protein and 1 μg/ml metalloprotease lethal factor mix, 16 h) or flagellin (100 ng recombinant protein mixed with PULSin, 16 h). Human monocytes were isolated from fresh peripheral blood mononuclear cells (PBMCs) using the EasySep Human Monocyte Enrichment Kit without CD16 Depletion (StemCell Technologies) following the manufacturer’s instructions. Alternative NLRP3 inflammasome activation was carried out by incubation of isolated human monocytes with 1 µg/ml of LPS during 4 h.

Human EEA1 construct^56^ was sub-cloned into pcDNA3.1/V5-His TOPO vector (Life Technologies) with a Nt c-Myc and Ct V5 epitope tags. EEA1 D127A and D132A mutations were incorporated by overlapping PCR. The full coding region of the constructs used in this study were verified by DNA sequencing. EEA1 expression vector was transfected into HEK293 cells with Lipofectamine 2000 according to the manufacturer’s instructions (Life Technologies).

### Amino terminal sequencing of cleaved EEA1

Over expressed recombinant human EEA1 was extracted from transfected HEK293 cells by incubation in cold lysis buffer (50 mM Tris-HCl, 150 mM NaCl, 1% NP-40; pH 7.5). After centrifugation, the supernatant was incubated with anti-V5-tag mAb-Magnetic Beads (MBL International Corporation) for 3 h at 4 °C. Magnetic beads were isolated and washed on a magnetic rack, and then, suspended in ICE buffer (0.1 M HEPES, 0.1% CHAPS, 10% Sucrose, 2.9 mM DTT; pH 7.5) with 1 U recombinant human caspase-1 for 2 h at 37 °C. Magnetic beads were then isolated, washed and suspended in 40 µl of reducing loading sample buffer (Invitrogen), boiled and subjected to SDS-PAGE. Gels were blotted to a PVDF membrane and proteins were revealed by Coomassie brilliant blue protein staining (BioRad). Cleaved EEA1 was cut and sent to Protein Chemistry Laboratory of the CIB-CSIC (Madrid, Spain), where it was processed and subjected to N-terminal sequencing by Edman’s sequential degradation in a Procise 494 protein sequencer (Perkin Elmer).

### *Eea1* gene silencing

For *Eea1* gene silencing, bone marrow cells were infected with short hairpin RNA (shRNA) lentiviral transduction particles, and experiments were performed after 7 days of macrophage differentiation. In brief, 10^6^ of bone marrow cells were plated in 6 well plates (day 0). At day 3, medium was removed and new medium containing 8 µg/ml hexadimethrine bromide (Sigma-Aldrich) and 10 MOI of lentiviral particles (EEA1 specific or non-target control) were added. On next day, medium with 3 µg/ml of puromycin was added, and cells were used at day 7.

### Immunocytochemistry and microscopy

Macrophages stimulated on cover slips were washed twice with PBS, fixed with 4% formaldehyde/4% sucrose in PBS for 5 min at room temperature, and then washed three times with PBS. Cells were blocked with 1% bovine serum albumin (Sigma) and permeabilized with 0.1% triton X-100 in PBS for 30 min at room temperature before incubating with primary antibody. Primary antibodies used were anti-EEA1(1:1000, BD Biosciences), anti-LAMP1 (1:1000, Santa Cruz Biotechnology), and anti-Rab5 (1:500, Cell Signalling). Primary antibodies were incubated for 16 h at 4 °C. Cells were washed and incubated with appropriate fluorescence-conjugated secondary antibody (1:200) for 2 h at room temperature, then rinsed in PBS. To stain for active caspase-1, macrophages were incubated with the fluorophore inhibitor of caspase-1, green fluorescent peptide 5-carboxyfluorescein-Tyr-Val-Ala-Asp-fluoromethyl ketone (FAM-YVAD-fmk, FLICA), according to the manufacturer’s recommendations (Immunochemistry Technologies). Labeling lysosomes with LysoTraker Red DND-99 (Life Technologies) was performed following manufacturer’s recommendations. In both protocols, cells were fixed with 4% formaldehyde, blocked, permeabilized and co-stained for EEA1 or LAMP1 as described above. All coverslips were mounted on slides with Fluoroshield with DAPI (Sigma-Aldrich). Images were acquired either with a Delta Vision RT (Applied Precision) restoration microscope using a Coolsnap HQ (Photometrics) camera, with a 60×/1.42 Plan Apo or 100×/1.40 Uplan Apo objectives, a Z optical spacing of 0.2 μm and the 360 nm/475 nm, 490 nm/528 nm and555nm/617 nm filter sets (Chroma 86000v2). Raw images were deconvolved using Softworxor ImageJ (NHI) software and maximum intensity projections of these deconvolved images are shown in the results. Co-localization analysis was performed using the Coloc 2 plugin for ImageJ (NHI).

### Co-immunoprecipitation and Western blotting

Detailed methods used for Western blot analysis have been described previously^24^. For co-immunoprecipitation, equal amounts of cell supernatant were incubated with 1.5 µg of anti-EEA1 or normal mouse IgG_1_(1 h, 4 °C) and then with protein-G-agarose beads (Merk-Millipore, 1 h, 4 °C). Beads were washed and heated (5 min, 80 °C) with reducing loading sample buffer (Invitrogen).

### *In vitro* EEA1 processing

Transfected HEK293T cells were lysed on hypotonic cell lysis buffer (25 mM HEPES pH 7.5, 5 mM MgCl_2_, 5 mM EDTA, 5 mM DTT) and lysates were incubated for 30 min at 37 °C with 1U of recombinant caspase-1 (Merck-Millipore), concentrate supernatants or cell lysates from nigericin treated macrophages.

### IL-1β ELISA

IL-1β release was measured by ELISA for mouseIL-1β (R&D Systems) or human IL-1β (eBiosciences) following the manufacturer’s instructions and read in a Synergy Mx plate reader (BioTek). The concentration of IL-1β was estimated using a standard with known concentrations of recombinant IL-1β.

### Lactate dehydrogenase (LDH) release and Yo-Pro-1 uptake

LDH release was measured using the Cytotoxicity Detection kit (Roche) following the manufacturer’s instructions, and expressed as percentage of total cell LDH content. Yo-Pro-1 uptake was performed as already described^9^.

### Statistical analysis

Statistics were calculated using Prism software (GraphPad). Comparisons of two groups were analyzed using two-tailed *t-*test and comparisons of multiple groups were analyzed by mean of analysis of variance (ANOVA) with Bonferroni’s post-test.

The datasets generated during and/or analysed during the current study are available from the corresponding author on reasonable request.

## Supplementary information

Supplementary Information

## Data Availability

Complete plots for Figs 1–4 are presented in Supplementary information. Other data are available from the corresponding author upon reasonable request.
