# Author Correction: Early endosome autoantigen 1 regulates IL-1β release upon caspase-1 activation independently of gasdermin D membrane permeabilization

**DOI:** 10.1038/s41598-020-78309-y

**Published:** 2020-12-01

**Authors:** Alberto Baroja-Mazo, Vincent Compan, Fátima Martín-Sánchez, Ana Tapia-Abellán, Isabelle Couillin, Pablo Pelegrín

**Affiliations:** 1 Inflammation and Experimental Surgery Unit, Biomedical Research Institute of Murcia IMIB-Arrixaca, Clinical University Hospital Virgen de la Arrixaca, 30120 Murcia, Spain; 2 Institute of Functional Genomics, Labex IC ST; INSERM U661, CNRS UMR5203, University of Montpellier.141, 34094 Montpellier cedex 5, France; 3 Molecular and Experimental Immunology and Neurogenetics, NEM, CNRS, UMR7355, University of Orleans, 45071 Orleans, France

Correction to: *Scientific Reports* 10.1038/s41598-019-42298-4, published online 08 April 2019

The Acknowledgements section in this Article is incomplete.

“We thank to Ana Isabel Gomez-Sánchez and Maria Carmen Baños (IMIB-Arrixaca, Murcia, Spain) for technical assistance with both molecular biology analyses and cell culture. Dr. Veit Hornung (Gene Center of the Ludwig-Maximilians Universität Munich, Munich, Germany) for caspase-1 and caspase-4 deficient THP-1, and Dr. Angel Nebreda (Institute for Research in Biomedicine, Barcelona, Spain) for EEA1 expression vector.”

should be:

“We thank to Ana Isabel Gomez-Sánchez and Maria Carmen Baños (IMIB-Arrixaca, Murcia, Spain) for technical assistance with both molecular biology analyses and cell culture. Dr. Veit Hornung (Gene Center of the Ludwig-Maximilians Universität Munich, Munich, Germany) for caspase-1 and caspase-4 deficient THP-1, and Dr. Angel Nebreda (Institute for Research in Biomedicine, Barcelona, Spain) for EEA1 expression vector. P.P. was funded by European Research Council, Grant/Award number: ERC-2013-CoG 614578.”

