# Supplementary material for: Early endosome autoantigen 1 regulates IL-1β release upon caspase-1 activation independently of gasdermin D membrane permeabilization
Source: Sci Rep. 2019 Apr 8;9:5788. doi: 10.1038/s41598-019-42298-4 (PMC6453936; doi:10.1038/s41598-019-42298-4)

SUPPLEMENTARY MATERIALS FOR:

Early endosome autoantigen 1 regulates IL-1β release upon caspase-1 activation independently of gasdermin D membrane permeabilization

Alberto Baroja-Mazo, Vincent Compan, Fátima Martín-Sánchez, Ana Tapia-Abellán, Isabelle Couillin and Pablo Pelegrín

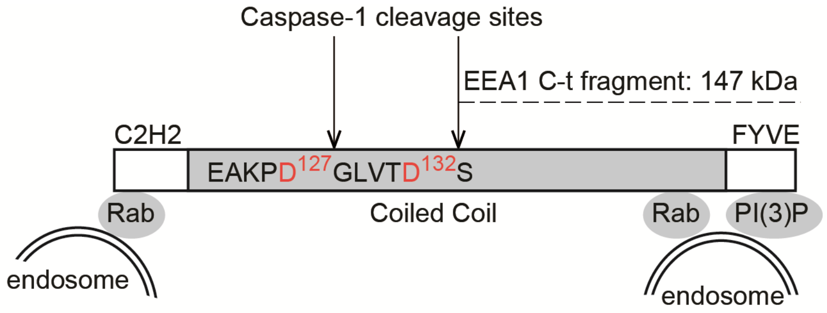

Supplementary Figure 1. Diagram representation of human EEA1 caspase-1 processing sites.

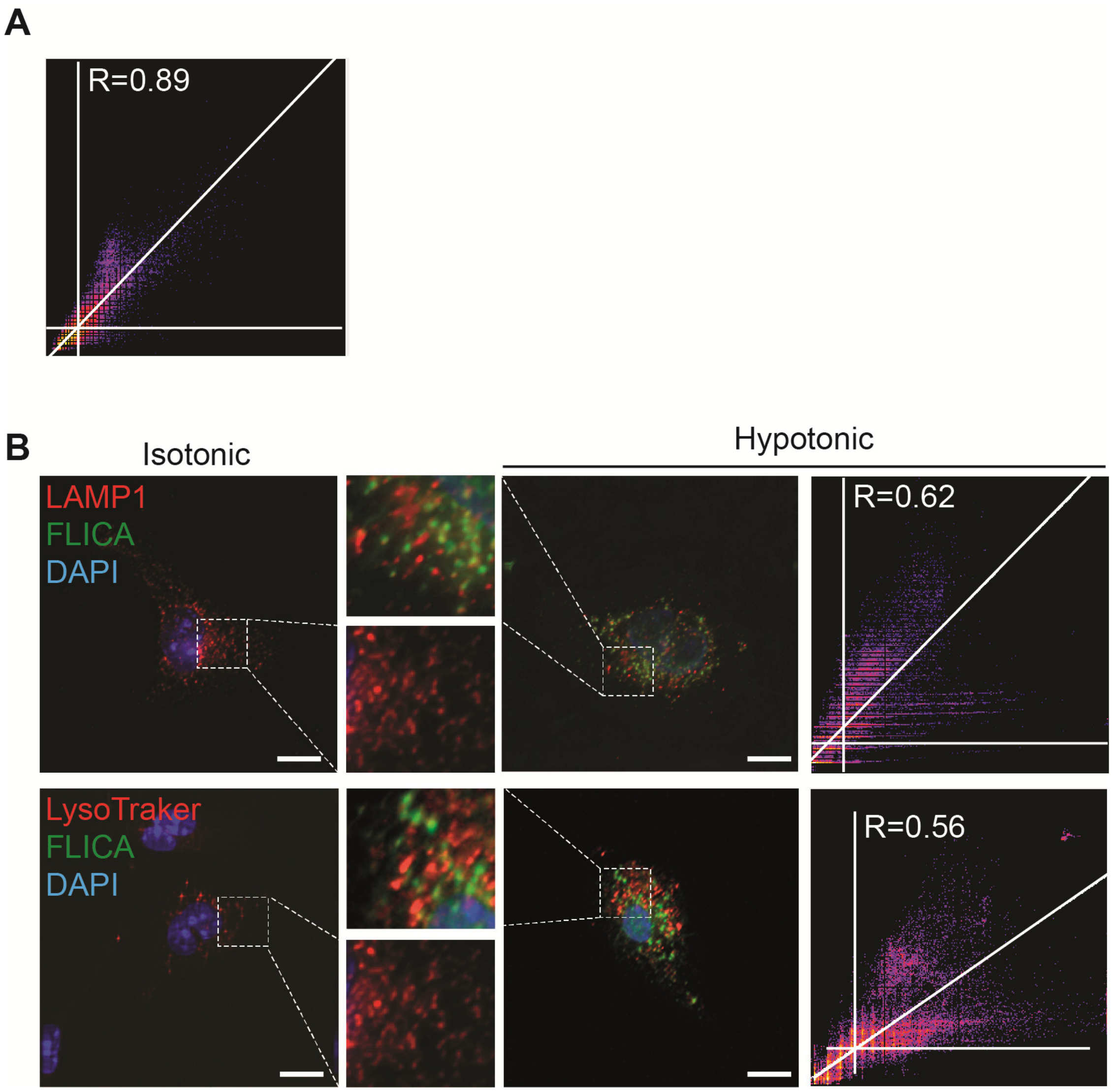

Supplementary Figure 2. (A) Co-localization regression of active caspase-1 stained with FLICA and endosomal marker EEA1 in LPS-primed BMDMs after canonical NLRP3 stimulation with hypotonicity, pictures are presented in figure 1E. **(B)** LPS-primed BMDMs after canonical NLRP3 stimulation with hypotonicity and stained with fluorescent probe for active caspase-1 (FLICA, green) and the lysosome markers LAMP1 or Lysotraker (red) and nuclei (DAPI, blue); bar, 5 μm. Panels on the right represent co-localization regression.

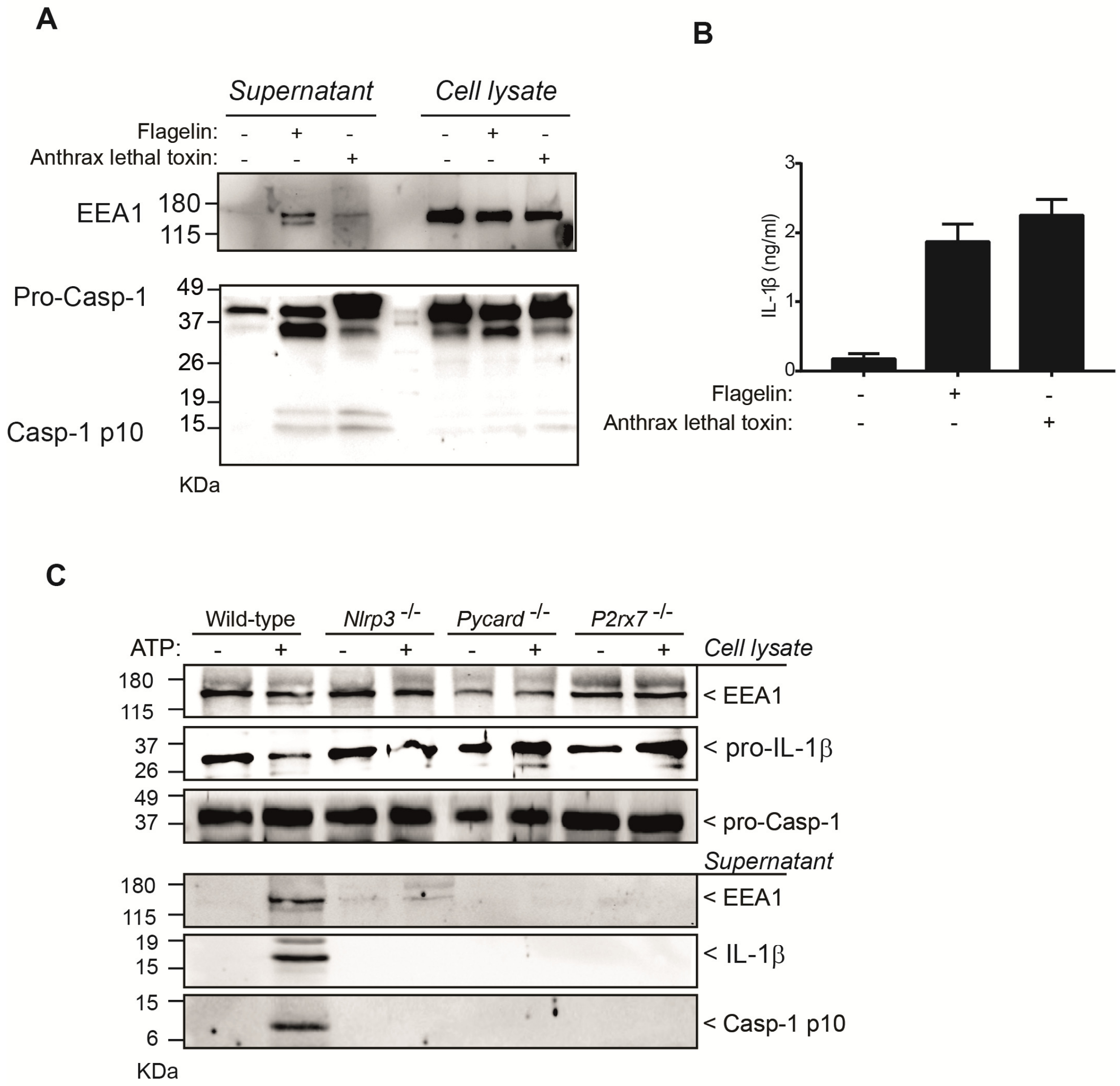

Supplementary Figure 3. **(A)** Immunoblot analysis of EEA1 and caspase-1 on cel lysate and cell-free supernatant from LPS-primed BMDMs after stimulation of NLRC4 inflammasome with flagelin and NLRP1 inlfammasome by anthrax lethal toxin. **(B)** IL-1β released to the extracellular medium as detected by ELISA. **(C)** Immunoblot analysis of EEA1, IL-1β and caspase-1 on cell lysate and cell-free supernatant from LPS-primed BMDMs from wild type *P2rx7-/-*, *Nlrp3-/-*and *Pycard-/-*after stimulation with ATP. Blots are representative of at least four independent experiments.

Supplementary Blots
Figure 2A
Pro-IL-1beta Cell Extract

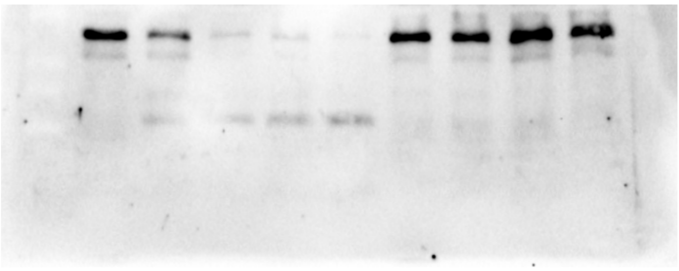

< Pro-IL-1beta

< IL-1beta

IL-1beta Supernatant

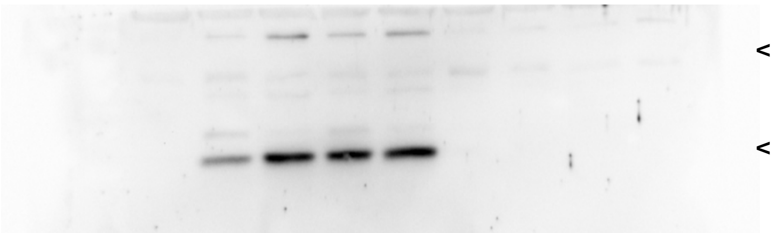

< Pro-IL-1beta

< IL-1beta

Figure 2D
Caspase-1 Supernatant

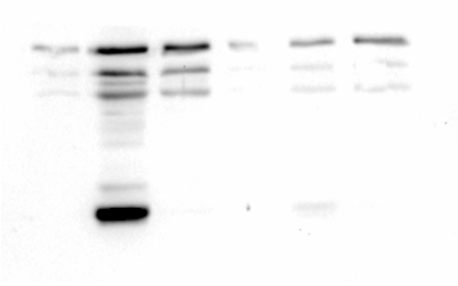

< Pro-Casp1

< Casp1 p10

IL-1beta Supernatant

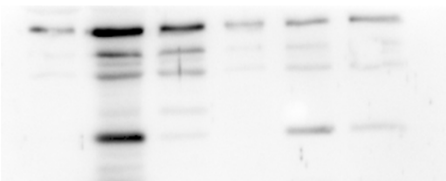

< Pro-IL-1beta

< IL-1beta

Figure 3A
Caspase-1

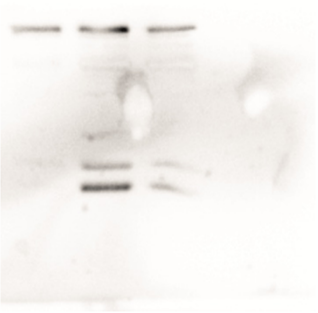

< Pro-Casp1

< Casp1 p10

IL-1beta

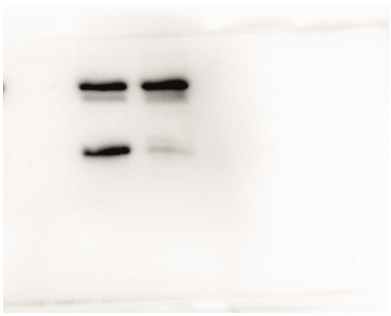

< Pro-IL-1beta

< IL-1beta

Figure 4A

Caspase-1 cell extract

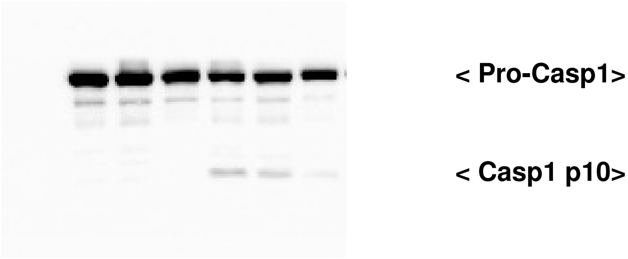

Caspase-1 supernatant

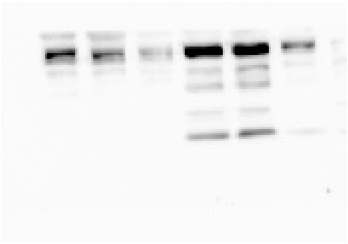

IL-1beta cell extract

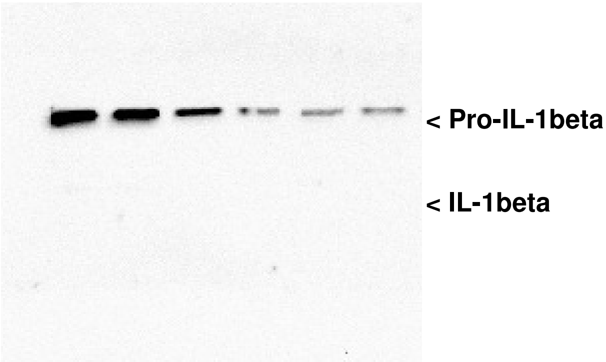

IL-1beta supernatant

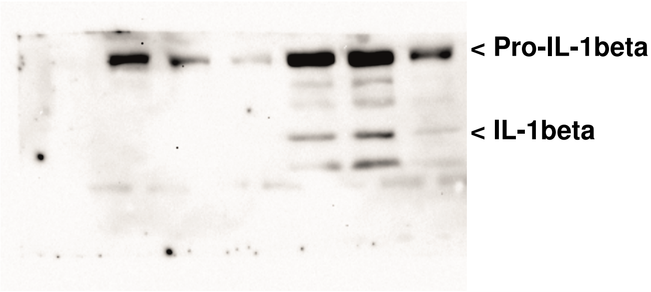

Supplement: Supplementary file 1 — Supplementary Information [file 41598_2019_42298_MOESM1_ESM.pdf]
